# The Progress of Comparative Pathology as Regards Infectious and Other Diseases

**Published:** 1883-04

**Authors:** 


### EDITORIAL DEPARTMENT.

### THE PROGRESS OF COMPARATIVE PATHOLOGY AS REGARDS INFECTIOUS AND OTHER DISEASES.

WE propose to give a summary of some of the remarkable advances which have been made in comparative pathology during the past two years, paying especial attention to the discoveries regarding the nature of certain of the infectious diseases affecting the domestic animals and man. We acknowledge indebtedness in part of this review to the work of M. C. Leblanc, contributed to the *Archives Generales de Medicine.*

*Anthrax.*—This disease has long been known to be caused by the anthrax bacillus. Pasteur and Davaine have made claims that the bacilli can be weakened in power and that then when inoculated the animals become protected. We have already described the methods in the JOURNAL. The question now is, are they really useful?

Pasteur gives some very convincing figures in support of the affirmative. On the other hand there have been failures reported in England and Italy.

The facts at present are these:

(1.) Immunity from anthrax can undoubtedly be given by inoculations.

(2.) The duration of this immunity is considerable (two years in some cases), but we do not yet know it to be permanent.

(3.) The practical usefulness of anthrax inoculations upon sheep and cattle has not yet been demonstrated. Very favorable statistics are given, but, on the other hand, the weather in France has been very moist for the past year and unfavorable to the production of anthrax so that these statistics are not entirely to be trusted.

(4.) The protective virus furnished by Pasteur and his assistants varies in quality. That used in 1882 was weaker in protective power and caused numerous accidents. The art of preparing protective anthrax virus is still imperfect.

*Infectious Pneumo-Enteritis of the Pig (Rouget de Porc), (Hog Cholera).*—This disease has a certain analogy to anthrax.

Dr. E. Klein claims to have discovered a specific micro-organism for it. M. LeBlanc denies that this is the real microbe, however, but avers that Pasteur has found it. The parasite in question is very minute, of a dumb-bell shape, refracts light very poorly and is not easily observed. It has, however, been isolated, cultivated, attenuated and a protective vaccine obtained. This vaccine has given sufficient results to demonstrate the scientific fact that it really possesses protective power.

It will be of the greatest importance to America if a protective vaccine can really be found since our country loses millions of dollars annually from "hog-cholera."

*Hydrophobia.*—The study of this disease by French savants is still going on. A micro-organism has not yet been discovered. A point, however, has been gained. Pasteur has found that the virulence lies not only in the saliva, but also in the cerebral and medullary substance. A portion of this substance placed, after trepanning, in contact with the brain of experimented dogs has caused the symptoms of hydrophobia to be developed and that in a time, relatively short compared with the duration of usual incubation after the biting or inoculation of the infected saliva.

All the animals did not die and one of them after having shown symptoms of the disease, became stubborn to the inoculation of the rabies. The question is still to be further studied. It is little probable that the process of trepanning can be adopted as a preventive measure, and it is to be hoped that more simple and practical means will be discovered.

Rabies is one of the contagious diseases, the symptoms, lesions and progress of which differ most widely from those of anthrax. It has no microbe and the blood is not virulent. It will be difficult then to attenuate the virus and to make preventive inoculations.

*Contagious Pleuro-pneumonia.*—The pathology and prevention of this disease is still the subject of discussion. Thirty years ago Dr. Willems claimed that inoculation was an infallible preventive, and his views have still many upholders. Recent experience has demonstrated the fallacy of this view. Holland, in 1881, adopted the policy of stamping out the disease in all but a few districts. The result was that the number of cases has fallen from over 2,000 in 1878 to 12 in 1881. On the other hand, in the districts where inoculation was employed the total loss amounted to 26 per cent.

The weight of knowledge and experience is, therefore, at present against the practice of inocculation. It is one which has never been attempted in America and probably never will be.

As to the pathology of the disease, it has no micro-organism according to Pasteur, whom we can trust more confidently when he makes such denials. He says in his report:

"I have ascertained, first, that the pleuro-pneumonia virus obtained from lungs diseased is a pure product not associated with germs of foreign microbes. Second, that the virus is not grown in our usual broths of chicken and veal, nor in the leaven of beer. Third, that the contrary conclusions obtained in Belgium are the results of error in manipulation."

Pasteur has collected the pleuro-pneumonic virus in a pure condition and he found that it was preserved in a very high temperature without production of miroscopic foreign organisms. Virus collected in the usual way becomes disturbed in twenty-four hours and develops foreign organisms.

Experiments with the pure virus upon cows show that it is really more dangerous than the impure and that danger lies in the strength, not the impurities. M. Leblanc finds that by using fresh virus in clean vessels the deaths from the inoculation of 1,000 cows were only two.

*Equine Variola and Glanders.*—Prof. M. Trasbot, of Alfort, has claimed that the above affections were identical, and that the glanders of horses might perhaps be prevented by vaccination.

M. Leblanc experimented with inoculations of human and bovine virus upon over one hundred horses. He found that although many developed pustules glanders was *not* prevented.

*The Germ of Farcy.*—Dr. Stivick, of Berlin, has discovered a minute organism resembling the bacillus of tuberculosis in the nodules of farcy. He cultivated it after Koch's method. The fourth generation was injected into two horses and produced farcy. They both died, and a post mortem showed the lesions of the disease.

*The Bacillus of Tuberculosis.*—Dr. R. Koch's discoveries in relation to phthisis have already been described in the JOURNAL.

*Typhoid Fever in Horses.*—This affection is rather infectous than contagious, and up to the present time veterinarians have not succeeded in transmitting it by inoculation. Analysis of the blood and its examination under the microscope have given contradictory results.

The disease is the result of overcrowding and change of diet. It is best dealt with by isolation and perhaps killing the affected animals. A preventive vaccine need hardly be wished for.

*The Rot or Ovine Variola (La Clavelée.)*—This disease is not infrequent in this country. Prof. Peuch, of Toulouse, has recently studied it. For some years it had been treated by inoculation in France. The results were very like those which followed inoculation for pleuro-pneumonia. Inoculation of the rot-virus, or "clavelization," however, differed in that it *always* produced the disease. The results had been so unfavorable that farmers preferred taking the chances of contagion to those of inoculation.

Prof. Peuch sought the means of diminishing the losses caused by the inoculation of the more diluted virus, and he has tried to inject it, after dilution, into the cellular tissue. The dilution has varied from a twentieth to a fiftieth degree.

All of the animals experimented upon lived, but, unfortunately, it is not yet certain that the diluted virus, though safer, really protects.

Prof. Peuch is continuing his investigations. If he succeeds the question is raised whether by a like process of dilution the virus of human variola might not be made protective.

*Remittent Ophthalmia of Horses, or Periodic Fluxion of the Eyes. (Moon Blindness.)*—This disease, which almost invariably destroys the eye attacked, has been the subject of a memoir by Messrs. Hocquard and Bernard.

These authors arrive at the conclusion that the periodic fluxion is a peculiar form of rheumatic diathesis such as effects all animals under the influence of moist surroundings, and is transmitted hereditarily.

The pathology of the disease is said to be an irido-choroiditis. The authors appear to be unaware that all which they announce as new was described in the JOURNAL OF COMPARATIVE MEDICINE for April, 1881, by Prof. W. O. Moore. No acknowledgement of the prior work of this gentlemen is given.

Certain German observers have been trying to show that the disease in question is of parasitic origin. Dr. Bertin, of Stuttgard has found in an affected eye a microscopic body of a fungoid character.

M. Kryslowicz has made a similar discovery. Dr. Koch, of Berlin, has found very minute organisms in the aqueous and and vitrious humor of the diseased eyes and also in the water of localities where this affection prevails. He has cultivated them and found that they had no specific powers of any kind· M. Zundel, however, still contends that the parasitic origin of the disease is undoubted. Our opinion is that the disease has nothing more specific about it in the horse than in other animals.

*An Epileptiform Disease in the Dog.*—In 1881 M. Mégnin, who is well-known for his works on helminthology, described a case of epilepsy in the dog caused by an acarus (tick) of the ear. According to him this acarus was the *chorioptes ecaudatus* already met with in the cat and ferret. This parasite provokes true attacks of mania in the cat, but it was the first time that its presence in the dog had given rise to accesses simulating epilepsy. M. Nocard, Professor at the Alford school, in 1882 read a note on an epileptical disease in the hunting dog, due to the presence in the ear of this same parasite, which Hering had taken for a *sarcopte* and of which Zurn had first given a description in 1874. The attacks in hunting dogs always come on during the chase, at the end of about half an hour.

The animal howls, turns in a circle two or three times, then falls a prey to an attack simulating epilepsy. He remains half stupified from fifteen to thirty minutes, then resumes his course without giving any other sign of sickness. The animal, howevsr, often becomes sad, fearful and savage.

At the autopsy there is found in the external auditory canal a mass of chocolate-colored wax of the consistence of mastic, obstructing all the light and driving back the membrana tympani. One finds under the microscope a quantity of ticks of all degrees of development and of different sexes. The disease is easily cured by daily injections of oil containing some parasiticide.

*The Rouget in Dogs.*—The skin diseases of dogs are numerous and but ill-understood. One of these, the *rouget*, is often confounded with the mange, but is really a very different disease and is probably of a diathetic nature. It begins in the groins and extends to the stomach, breast and inner surface of the legs, but never to the back or loins, in this respect being distinct from the mange. The skin in this disease, according to Mégnin, is studded with superficial punctiform erosions, the remains of vesicles. In some places there is an oozing out of pus. The disease resembles eczema rubrum in man. The mange is often called an eczema by veterinarians, but wrongly. The best treatment is arsenic internally, gr. $\frac{1}{10}$, daily, raw meat. Externally, weak carbolic acid lotions and antiseptic baths are recommended.

*Vaginal Cystocle with Displacements of Bladder and Uterus in the Cow.*—This trouble is not a rare one, and Prof. Violet, of Lyons, has contributed some useful facts regarding it.

The vaginal cystocle of cows is often the consequence of the retroversion of the uterus. The bladder, dragged by the walls of this organ is found carried to the side of the vulva, where it sometimes breaks through the orifice. Often the relief of the uterine retroversion is very difficult by reason of the displacement of the bladder. Once this displacement is recognized, however, and the organ emptied by the aid of a catheter, the replacement becomes easy.

*Perineal Cystocele in the Dog.*—Perineal cystocele is frequent in the dog. The veterinarian will recognize it when he sees a semi-ovoid tumor, soft and fluctuating below the anus on the circumference of the ischial arch.

The size may vary but it is generally about that of a hen's egg. The most frequent cause is constipation, and the animal does not complain for a long time of any pain, nor does any symptom of indiposition warn his master until the bladder becomes irritated and there is retention of urine. As a catheter cannot be introduced an opening must generally be made with a trochar and the urine withdrawn. The bladder is then replaced. The trouble has a tendency to recur.

*Lymphadenoma in the Dog. A New Disease.*—This very rare affection has been discovered for the first time by M. Nocard. The skin of the animal affected is covered with nodules varying in size from that of a pea to that of a nut. The surface is red and granular.

There is evidently a hypertrophy of the papillæ of the skin which have lost their epidermis. The skin is thickened. On its surface a very albuminous liquid is seen to issue, having a very fetid odor. In other parts of the skin small hard bodies about the size of a pea is felt. The general state of the animal is that of progressive weakness. There is no sugar or albumen in the urine. There is a slight diminution of the red globules and an almost normal number of the white. The liquid of the spots was inoculated in a young dog and two kittens without any success.

Under the microscope a layer of adenoid tissue was found developing in all the elements of the skin and growing with great vigor.

*American Work.*—It is but due to the JOURNAL OF COMPARATIVE MEDICINE to say that during the past three years it has contributed a large amount of original work. *The Pathology of Posterior Paralysis* has been well elucidated by Dr. W. H. Porter, Dr. C. A. Meyer and Dr. G. W. Bowler.

*The Pathology of Moon-Blindness* was first described in this Journal by Professor W. O. Moore.

Original observations upon the *Sterility of Mammalia Under Confinement* have been made by Mr. Arthur Erwin Brown.

To the subjects of *Comparative Physiology and Psychology*, Dr. Wm. A Hammond and the late Dr. George M. Beard both have made valuable contributions. The results of original studies upon *Animal Parasites* and upon other branches of pathology have been given by Dr. T. E. Satterthwaite.

A very large number of contributions to *Morbid Anatomy* have been made by Professor Porter and Dr. E. C. Wendt. In no other veterinary journal in any language presents the results of so much careful microscopical work.

